# Sex ratio affects sexual selection against mutant alleles in a locus-specific way

**DOI:** 10.1093/beheco/arad110

**Published:** 2023-12-29

**Authors:** Sakshi Sharda, Brian Hollis, Tadeusz J Kawecki

**Affiliations:** Department of Ecology and Evolution, University of Lausanne, Biophore, CH-1015 Lausanne, Switzerland and; Department of Biological Sciences, University of South Carolina, 715 Sumter St., Columbia SC 29208, USA; Department of Ecology and Evolution, University of Lausanne, Biophore, CH-1015 Lausanne, Switzerland and

**Keywords:** *Drosophila*, female choice, good genes, genic capture, male–male competition, opportunity for sexual selection, purging of deleterious mutations

## Abstract

Higher male:female operational sex ratio (OSR) is often assumed to lead to stronger sexual selection on males. Yet, this premise has been directly tested by very few studies, with mixed outcomes. We investigated how OSR affects the strength of sexual selection against two deleterious alleles, a natural *ebony* mutant and a transgenic *GFP* insertion, in *Drosophila melanogaster.* To this end, we estimated the relative paternity share of homozygous mutant males competing against wild-type males under different OSRs (1:2, 1:1, 2:1). We also manipulated the mating pool density (18, 36, or 54 individuals) and assessed paternity over three consecutive days, during which the nature of sexual interaction changed. The strength of sexual selection against the *ebony* mutant increased with OSR, became weaker after the first day, and was little affected by density. In contrast, sexual selection against the *GFP* transgene was markedly affected by density: at the highest density, it increased with OSR, but at lower densities, it was strongest at 1:1 OSR, remaining strong throughout the experiment. Thus, while OSR can strongly affect the strength of sexual selection against “bad genes,” it does not necessarily increase monotonically with male:female OSR. Furthermore, the pattern of relationship between OSR and the strength of sexual selection can be locus-specific, likely reflecting the specific phenotypic effects of the mutation.

## INTRODUCTION

Sexual selection is a major force driving the evolution of morphological and behavioral diversity. Studying factors that affect the strength of sexual selection can contribute to understanding patterns of this diversity, as well as of other evolutionary consequences of sexual selection, such as trade-offs associated with the development and maintenance of sexual traits (Rowe and Houle 1996; Bonduriansky and Chenoweth 2009), the degree of sexual dimorphism (Bro-Jorgensen 2007; Kelly 2008; Hollis et al. 2014; Grath and Parsch 2016; Hamalainen et al. 2018), sexual conflict (Chapman et al. 2003; Bonduriansky and Chenoweth 2009), speciation (Maan and Seehausen 2011), and ecological processes (Giery and Layman 2019).

A major factor hypothesized to influence the strength of sexual selection is the operational sex ratio (OSR), that is, the relative numbers of males and females interacting in the context of potential mating at a given time. The strength of sexual selection on males has been proposed to increase with increasing OSR (males to females) (Emlen and Oring 1977; Clutton-Brock and Parker 1992; Kvarnemo and Ahnesjo 1996; Clutton-Brock 2017; Janicke and Morrow 2018). (An analogous prediction applies to females in species where females compete for males (Emlen and Oring 1977), but in the present paper, we focus on sexual selection on males.) This prediction is based on two assumptions: (1) that stronger competition for mates resulting from higher OSR increases the opportunity for sexual selection (*I*_*S*_), and (2) that greater *I*_*S*_ results in stronger sexual selection (Klug et al. 2010; Krakauer et al. 2011).

Both these assumptions have been questioned. First, a universal positive relationship between OSR and *I*_*S*_ is not predicted from theory (Klug et al. 2010; Krakauer et al. 2011; Jennions et al. 2012; Kokko et al. 2012). Empirically, though, many studies do report a positive correlation between the two within a species (e.g., Cade and Souroukis 1993; Jirotkul 2000; Jones et al. 2004; Croshaw 2010; Wacker et al. 2013; House et al. 2019), and a positive correlation has been found across species (Janicke and Morrow 2018). Second, even though *I*_*S*_ sets the upper limit to the strength of sexual selection, realized sexual selection on any trait or any genetic polymorphism is, in general, not predicted to correlate with *I*_*S*_ (Klug et al. 2010). And, in contrast to the first assumption, such a correlation is generally not supported by empirical data (Klemme et al. 2007; Klug et al. 2010; Jennions et al. 2012). Yet, much of the interest in sexual selection is about understanding how it favors specific traits (such as costly weapons or ornaments) or gene variants (“good genes”).

The strength of sexual selection on specific traits or genetic variants is more directly addressed by quantifying their relationship with sexual components of fitness in terms of selection differential, selection gradient, or selection coefficient (Arnold and Wade 1984; Klug et al. 2010). Only a few studies have taken this approach to testing the effect of OSR on the strength of sexual selection. Apparently, an increase in the strength of sexual selection on male phenotypic traits with OSR was only demonstrated with statistical support in one study (Wacker et al. 2013), with a couple of others reporting similar trends without statistically testing for them (McLain 1992; Cade and Souroukis 1993; Jones et al. 2005). Two other studies demonstrated a decline of selection on male traits with increasing OSR (Klemme et al. 2007; Fitze and Le Galliard 2008), and a few found no or inconsistent effects (Jones et al. 2004; Mills et al. 2007; Head et al. 2008). Similarly, in a sex-reversed fish species, the strength of sexual selection on body size in females is apparently not affected by sex ratio (Aronsen et al. 2013). Based on these few studies, a positive relationship between OSR and the strength of sexual selection on phenotypic traits seems to be more an exception than a rule. To our knowledge, no published study tested the effects of OSR on sexual selection against deleterious mutations (i.e., in favor of “good genes”).

Here, we address this gap by testing the effects of OSR on the strength of sexual selection against single mildly deleterious mutant alleles in *Drosophila melanogaster*, a promiscuous species subject to complex pre- and post-copulatory sexual selection (Ferveur 2010; Baxter et al. 2018; Lupold et al. 2020; Wigby et al. 2020) under variable and often strongly male-biased OSR (Atkinson and Shorrocks 1977; Soto-Yéber et al. 2019). Focusing on Mendelian polymorphisms rather than on quantitative traits provides a direct link to genetic consequences of female choice and changes in the gene pool. Furthermore, the “genic capture” theory of sexual selection proposes mildly deleterious mutations as the main source of genetic variation on which sexual selection acts (Rowe and Houle 1996). We used two mutant alleles, a spontaneous null mutant at the *ebony* gene and a chromosomal insert resulting in transgenic expression of green fluorescent protein (*GFP*) in the whole body and sperm. Both mutant alleles reduce male sexual competitiveness (see Methods). We let males homozygous for either mutant allele compete for females against wild-type males with the same genetic background under different OSRs. We quantified the paternity share of the two male genotypes, used it to estimate the sexual component of relative fitness of the mutants and tested how it is affected by the OSR treatments. With this approach, we directly test whether the overall strength of sexual selection, including its pre- and post-copulatory components, increases with male:female OSR.

Along with OSR, we manipulated the total number of interacting individuals (i.e., mating pool density). Differences in OSRs inherently imply a change in the number of at least one sex. Experimental evidence suggests that mating pool density can also affect sexual selection independently of OSR (McLain 1992; Jirotkul 1999; Holveck et al. 2015; Yun et al. 2018) or in interaction with it (House et al. 2019). Simultaneously manipulating OSR and density in a factorial design allowed us to disentangle the effects of these two factors. Furthermore, the rationale for the proposed effect of OSR is based on the numbers of the two sexes relative to each other, but an apparent effect of OSR could be driven by the absolute density of one sex in the mating pool (Fairbairn and Wilby 2001; Head et al. 2008; Wacker et al. 2013). To address this possibility, we analyzed the results in two alternative frames of reference, one using OSR and total density of both sexes as predictor variables of the relative success of *ebony* or *GFP* males, the other using male and female densities as predictors. This latter analysis would reveal if the effects of OSR and/or density were driven mainly by the density of one sex.

We quantified the relative sexual fitness of the mutant males over three consecutive days, starting with virgin individuals. During this period, the focus of sexual selection is likely to change from mate competition for/ mate choice by initially virgin females among naive males to sperm competition and its avoidance, mediated in part by seminal fluid proteins (Wigby et al. 2020). At the behavioral level, previously mated and experienced females not only become recalcitrant to remating but also more choosy (Dukas 2005b; Kohlmeier et al. 2021; Richardson and Zuk 2022), whereas males learn to be better at recognizing and courting receptive females (Dukas 2005a). Our design allowed us to explore how this changing mode of sexual selection affects its relationship with OSR.

## METHODS

### Fly strains and rearing

The experimental *D. melanogaster* strains were derived from a laboratory-adapted population called IV, established from wild flies collected in Amherst (Massachusetts, USA) in 1975 (Charlesworth and Charlesworth 1985) and maintained in the lab at high density with a census size in thousands. The mutant *ebony* strain was established in 1992 by backcrossing a spontaneous *ebony* null mutation into the IV population (Houle and Rowe 2003). The *ebony* gene codes for an enzyme involved in the regulation of biogenic amines such as dopamine, histamine, and melanin (https://flybase.org/reports/FBgn0000527.html). In addition to rendering adult cuticles darker than normal in homozygous state, the *ebony* mutation impairs vision and courtship, leading to a reduced mating success (Kyriacou 1985). The *ebony* strain we used here is inferior in terms of larval competition (Houle and Rowe 2003; Kawecki 2020), and males are less successful than wildtypes in achieving matings under sexual competition (Hollis and Kawecki 2014). However, they achieve the typical 80–90% paternity when the last male to mate (P2; Hollis et al. 2019), suggesting no major impairment in sperm competition. The *GFP* strain carries a genomic insertion that constitutively expresses two *GFP* transcripts, one under the control of a *ubiquitin* promoter active in all tissues throughout development and another under the control of *protamine B* promoter, specifically expressed in sperm cells (Manier et al. 2010; Lupold et al. 2012). For our study, the *GFP* construct was first backcrossed into the IV background for four generations. The *GFP*-mediated fluorescence enabled us to measure the relative paternity success of these males at the embryo stage, providing a more direct representation of competitive fertilization success (Lupold et al. 2012, 2020). The *GFP* insertion (at least in a different genetic background) reduces male success in sperm competition, notably in sperm defense, although not in sperm offense (Manier et al. 2010). It is not known, however, if it also affects precopulatory aspects of sexual selection, that is, mating success. As this may be useful in interpreting the effects of OSR on sexual selection, we performed competitive mating trials between *GFP* and wild-type males (see below).

All flies were reared on 2% w/v yeast media (water, agar [Milian CH], brewer’s yeast [Migros CH], cornmeal, sucrose, and Nipagin [Sigma-Aldrich CH]) and maintained on a 12L:12D photoperiod at 25 °C and a relative humidity of 55%. Virgin flies of both sexes were collected upon emergence and maintained in single-sex groups for 5–6 days before the start of the experiments, at which point the desired number of males and females were transferred to the experimental cages under light CO_2_ anesthesia.

### Measuring competitive paternity success

To address the main question of this study, we quantified the paternity share of homozygous mutant males competing against wild-type IV males under different OSR and numbers of the interacting individuals (density). Three OSR treatments (1:2, 1:1, and 2:1, males:females) and three density treatments (18, 36, and 54 total individuals) were combined in a factorial way. Statistical power to compare proportions is greatest around 0.5; yet, we expected the mutant males to be generally less successful than WT males. To compensate for this, irrespective of the treatment, the ratio of competing mutant to wild-type males was always 2:1. For example, the treatment with OSR 1:2 and a total density of 18 consisted of 4 mutant males + 2 wild-type males + 12 females. Because the *ebony* mutant phenotype is recessive, females used to assess this mutant’s paternity share were also homozygous for the *ebony* mutant allele; this way offspring sired by the mutant and wild-type males had mutant and wild-type phenotypes, respectively. In contrast, the *GFP* marker is dominant; thus, to assess the paternity share of *GFP* males, we competed them against wild-type males for wild-type females.

The experiments were performed in rectangular transparent polystyrene cages (L × W × H: 100 × 85 × 46 mm, volume 391 mL), with mesh-covered ventilation holes on the long sides (Supplementary Figure S1). Each cage had two circular inserts for replaceable Petri dishes (60 mm diameter) with food medium or medium for collecting eggs (agar + orange juice sprinkled with baker’s yeast). The medium in each Petri dish was additionally divided in two with a paper partition. This partitioning provided additional structure for females to potentially escape male harassment (Yun et al. 2018). The Petri dishes with the medium were replaced twice a day: fresh standard food was provided every morning (around 8:30) and was replaced by the oviposition medium in the evenings (17:00). The flies were transferred to the cages on the morning of day 0 and left there for 72 h. Each morning the 3 days of the experiment eggs laid overnight were collected and used to estimate paternity.

In the experiment with *GFP* males, we left the oviposition Petri dishes with eggs at room temperature for 8 h to give enough time for the embryos to develop sufficiently to allow unambiguous scoring of the *GFP* phenotype; the Petri dishes were then stored at 4 °C overnight. Embryos and any larvae that may have hatched were collected the next morning, pooled between the two Petri dishes, and suspended in water. The suspension was sub-sampled haphazardly, and the *GFP* versus non-*GFP* embryos and larvae were counted under blue light (440–460 nm). We targeted 50–70 embryos, but the subsampling was imprecise, and in some cases, fewer than the target number of eggs were available. As a consequence, the number of sampled embryos ranged from 28 to 118 (mean = 62, SD = 16).

Assessing paternity share of *ebony* males required raising the offspring to adulthood (the phenotype is not visible in embryos). Therefore, each morning the eggs were washed out of the oviposition medium, pooled between the two Petri dishes, and a haphazardly sampled batch of 45–60 eggs was transferred to a vial with fresh fly food (sometimes fewer when not enough eggs were available). When development was completed, the adults with wildtype and *ebony* phenotypes were counted. This means that the estimate of paternity share may be biased by differences in the survival of *ebony* and wild-type larvae. However, larval density was adjusted to the same approximate target in all treatments, and this larval density was low relative to the amount of food available. Therefore, the potential bias due to differential survival of *ebony* and wild-type larvae should be the same across treatments and thus should not confound the relationship between treatments and paternity share. Nonetheless, to make sure, we verified this statistically (see below).

We tested the effects of OSR and density on the sexual success of the two mutants in separate experiments, each comprising four experimental blocks spread over several weeks. The design was somewhat unbalanced in that we set up more replicates for the lower-density treatments, expecting them to be more affected by stochastic variation (8–16 per OSR × density combination for the *ebony* experiment and 10–11 for the *GFP* experiment). If more than a single fly (of either sex) died or escaped during the change of food dishes, the replicate was discontinued. Several further data points were lost for various reasons, resulting in varying sample sizes (Supplementary Table S1).

### Competitive mating trials of *GFP* males

We assessed the ability of *GFP* to acquire mates while in competition against wild-type males in a similar setting as in the paternity experiment—in the same cages, under mating pool density of 36 individuals, 1:1 sex ratio and with twice as many *GFP* as wild-type males (i.e., 12 *GFP* males + 6 wild-type males + 18 wild-type females). Because this was done under ambient light, under which the *GFP* and wild-type males are not distinguishable, we marked them with red and green color powder (Sennelier) in a balanced design, as described in Joye and Kawecki (2019). Virgin flies were introduced in the cages in the morning, and the number of matings by *GFP* and wild-type males was scored every 15–20 min for 8 h (*N* = 21 replicate cages).

### Statistical analysis

We performed all statistical analyses in R v3.4.3 (R Core Team 2021) with the package *afex* (Singmann et al. 2015), a wrapper for *lme4* (Bates et al. 2015). For the paternity experiments, we fit generalized linear mixed models (*glmer*) with the binomial distribution and logit link, where the response was the count of the mutant versus wild-type offspring in each replicate on each day.

We analyzed both experiments in two alternative reference frames: (1) using the log_2_ of the operational sex ratio and total density (males + females) as continuous explanatory variables and (2) using male and female densities as continuous explanatory variables. The day of egg collection was the third continuous explanatory variable in both frameworks. To facilitate model convergence and interpretation of the models, we centered and rescaled the total density values (18, 36, and 54 individuals), respectively to −1, 0, and 1. For the second reference frame, the numbers of males and females (each ranging from 6 to 42) were rescaled to range from −1 to 1. The 3 days were recoded as 0, 1, and 2; the log_2_ OSR variable is already centered on zero. With this rescaling, in either reference frame 1:1 sex ratio at density 36 on the first day of the experiment was an implicit “baseline” set of conditions (i.e., corresponded to the intercept of the model).

To account for potential confounding effects of variation in larval density in the *ebony* experiment (see above), we also fitted a model additionally including the total number of emergent offspring (scaled to mean = 0 and SD = 1) as a covariate.

All models initially included the experimental block and the replicate cage as random effects; the block was dropped from the analysis of the *ebony* experiment because the corresponding variance component tended to zero and caused model convergence issues. Significance was assessed with likelihood ratio tests, although *z*-tests produced essentially identical results. We first fitted a full model with all interactions (including three-way) between the explanatory variables. Interactions with *P* > 0.10 were subsequently dropped. Based on the patterns of significant interactions, we then analyzed the results separately for each day (for *ebony*) or for each density (for *GFP*). Quadratic factors were tested and retained if significant.

Note that the (sexual) fitness of mutant (*ebony* or *GFP*) males relative to wild-type males is

$Relative\;\;\;fitness\;\;\;of\;\;\;mutants=\frac{1}{2}\frac{Number\;\;\;of\;\;\;mutant\;\;\;offspring\;}{Number\;\;\;of\;\;\;wild\;\;\;type\;\;\;offspring\;};$ the division by 2 accounts for the 2:1 ratio of mutant to wild-type males. Thus, logit of the proportion of *ebony* offspring or *GFP* eggs equals ln(relative fitness of mutant males) – ln(2). Parameter estimates from our logit models can thus be directly interpreted in terms of differences in the natural logarithm of relative sexual fitness of the mutant males. Based on the same rationale, we also used the R package *emmeans* (Length 2022) to test if the overall mean of ln(mutant paternity share) estimated from the model deviates from ln(2). This was a way of testing if the relative fitness of the mutant averaged across the experimental conditions was different from 1, and thus if there was selection against (or for) the mutant. For the plots, we calculated the relative ln fitness of mutant flies for each replicate on each day and use those to calculate the mean and standard error for each OSR × density × day combination. For the above reasons, we also plot the relative fitness on a logarithmic scale; this is the scale biologically most relevant for fitness (Houle et al. 2011).

For the mating competition experiment, the numbers of matings were compared in a generalized mixed model assuming log link and Poisson distribution, with male genotype and color marking scheme as categorical factors, time from the onset of the experiment as a continuous explanatory variable and replicate as a random factor. Interactions were explored; nonsignificant interactions were removed from the model.

## RESULTS

### Sexual selection against *ebony*

**Figure 1:**
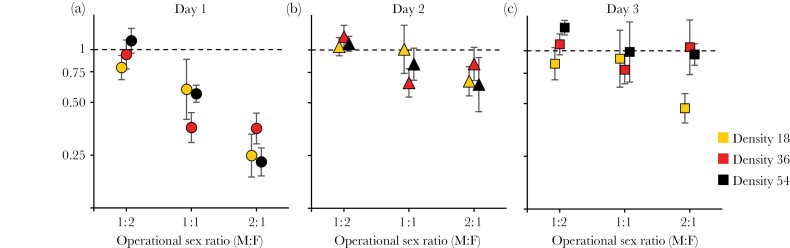
Relative sexual fitness of *ebony* males (i.e., paternity share per individual) under different conditions of operational sex ratio and density of the mating pool, based on offspring produced on the three consecutive days of the experiment. Symbols are means ± SE, *N* = 5–16 replicates per point.

The overall mean (± SE) log paternity share of *ebony* was 0.34 ± 0.05, significantly smaller than ln(2) = 0.69 (*z* = −6.9, *P* < 0.0001) and corresponding to the relative fitness of 0.70. The relationship between relative sexual fitness of *ebony* males and OSR and density changed over the course of the experiment (log OSR × day interaction χ^2^_1_ = 55.4, *P* < 0.0001, density × day interaction χ^2^_1_ = 14.1, *P* = 0.0002; Supplementary Table S2). Therefore, we analyzed the data from each day separately (Figure 1, Supplementary Figure S2). Even though the relative fitness of *ebony* males declined with OSR on each day, the relationship became less steep after the first day of the experiment (day 1: *b* = −0.57 ± 0.09, day 2: *b* = −0.23 ± 0.08, day 3: *b* = −0.21 ± 0.08; slope ± SE). Reflecting the density × day interaction, the relative fitness of *ebony* increased somewhat with density on day 3 (*b* = 0.26 ± 0.09, *P* = 0.0063) but not on day 1 and 2 (*b* = 0.03 ± 0.09 and *b* = 0.00 ± 0.08, respectively, both *P* > 0.75, Supplementary Table S2), no interaction between density and OSR was detected on any day (all *P* > 0.25, details not shown). Thus, even though sexual selection against the *ebony* mutant increased with the OSR, this relationship appeared to become less strong over the course of the experiment. However, given that at the female-biased sex ratio of 1:2 there was virtually no sexual selection against *ebony* on any day (relative fitness of *ebony* is essentially 1), the decrease in the slope of the relationship between OSR and *ebony* fitness could be explained by a general decrease in the strength of sexual selection against *ebony* after the first day of the experiment.

The mean total number of emergent offspring (*ebony* + wildtype) ranged across the treatment and day combinations from 40.3 to 49.2. Including the total offspring as a covariate had a negligible effect on parameter estimates and significance of OSR, density, and day (Supplementary Table S3). Thus, the conclusions about the effects of those factors on the relative sexual fitness of *ebony* males are not confounded by the potential effects of differences in larval density.

In the alternative frame of reference, with male and female density instead of OSR and total density as explanatory variables, the regression parameters for male and female densities were of opposite signs and similar in magnitude (male *b* = −0.53, female *b* = 0.50, male × day *b* = 0.28, female × day *b* = −0.17, all *P* < 0.001, Supplementary Table S4). In other words, the success of *ebony* decreased with male number and increased with female number with slopes of similar steepness. This confirms that it is the ratio of the males to females and not just the density of one sex that drives the relationship between OSR and the relative sexual fitness of *ebony* males.

### Sexual selection against *GFP*

**Figure 2:**
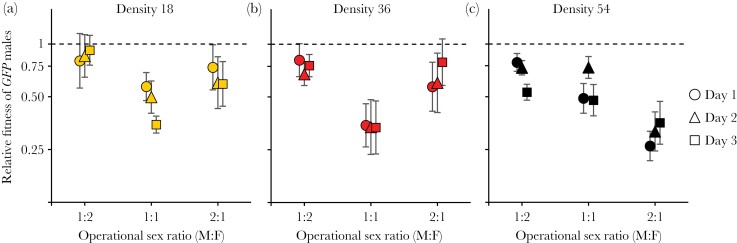
The effect of OSR and mating pool density on the relative sexual fitness of *GFP* males. Panels are split by density (rather than by the day of egg collection) as the pattern was affected more by density than by day. Symbols are means ± SE, *N* = 8–11 replicates per point.

The mean (± SE) of the natural log paternity share of the *GFP* males was 0.096 ± 0.145, significantly less than ln(2) (*z* = −4.1, *P* < 0.0001), indicating that across the experimental conditions their mean relative sexual fitness was 0.55. The full model indicated that the relationship between OSR and *GFP* male paternity share (and thus sexual fitness) was strongly affected by density (log OSR × density interaction, χ^2^_1_ = 7.0, *P* = 0.008), but did not change over the course of experiment (log OSR × day, χ^2^_1_ = 0.7, *P* = 0.40; Supplementary Table S5). Therefore, we analyzed and plotted the results separately for each density (rather than separately for each day as we did for the *ebony* experiment). At the highest density of 54 individuals (Figure 2c), the results resembled those for *ebony*: paternity share of *GFP* males declined monotonically with OSR (χ^2^_1_ = 22.5, *P* < 0.0001), and the slope of the relationship became less steep over the course of the experiment (log OSR × day χ^2^_1_ = 6.8, *P* = 0.0092, Supplementary Table S5). In contrast, the relationship between OSR and the relative fitness of *GFP* males under the two lower densities was distinctly nonlinear, the fitness being lowest at 1:1 sex ratio (Figure 2a,b). This nonlinearity is confirmed by significant quadratic OSR term for density 36 (log OSR^2^: χ^2^_1_ = 3.9, *P* = 0.047) and by the interaction between the quadratic OSR and day for density 18, consistent with curvature increasing over the course of the experiment (log OSR^2^ × day: χ^2^_1_ = 13.2, *P* = 0.0002; Supplementary Table S5).

In the alternative frame of reference, corresponding model parameters for male and female density were of opposite signs and similar magnitude (male *b* = −0.15, female *b* = 0.14, male × day *b* = 0.04, female × day *b* = –0.08), even though only this last parameter was significantly different from zero (Supplementary Table S6). Thus, as in the case of *ebony* males, the number of both sexes contributed similarly to the effects of OSR and total density on the strength of sexual selection against the *GFP* males.

### Mating success of *GFP* males

**Figure 3:**
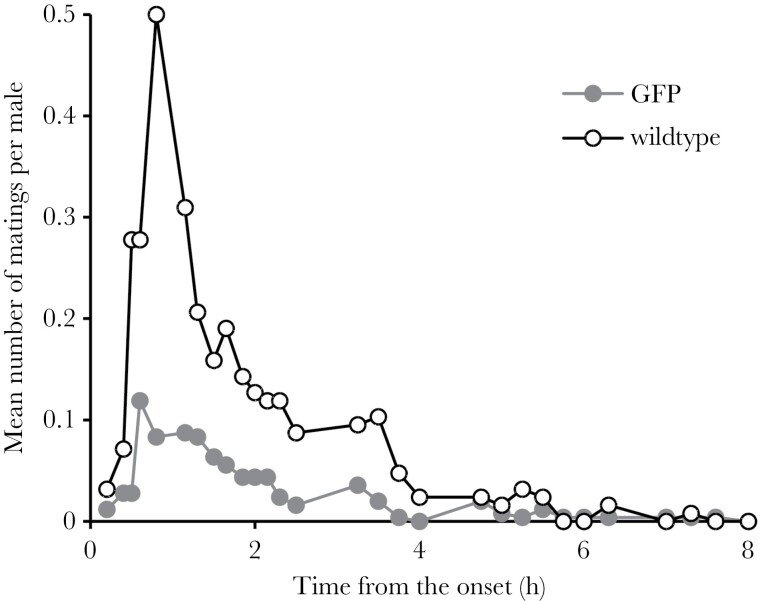
The average number of observed matings per *GFP* and per wild-type male over the course of the competitive mating experiment (*N* = 21 cages, each with 12 *GFP* males, 6 wild-type males and 18 wild-type females).

When competing with wild-type males under the conditions corresponding to 1:1 sex ratio and density of 36 individuals (12 *GFP* males, 6 wild-type males, 18 wild-type females), the *GFP* males were clearly less successful than the wild-type males in achieving mating. Throughout the 8 h of the experiment, the *GFP* males were recorded mating on average 10.3 ± 1.1 times, the wild-type males 18.0 ± 1.6 times, despite the former being twice as numerous (mean per replicate cage ± SE). While the numbers of matings declined over the 8 h of the experiment (Figure 3; χ^2^_1_ = 471.8, *P* < 0.0001, GLMM with log link and Poisson distribution), the ratio of matings by the two genotypes remained consistent (genotype: χ^2^_1_ = 44.5, *P* < 0.0001; genotype × time interaction: χ^2^_1_ = 1.6, *P* = 0.21). Marking color also had an effect with red-colored males achieving nearly 40% more matings than green-colored males (χ^2^_1_ = 15.8, *P* < 0.0001). Taking into account the fact that the *GFP* males were twice as numerous as the wild-type males, the mating success of *GFP* relative to wildtype was only 29%. We note that this is a value similar to the relative fitness of *GFP* based on paternity on the first day under the same density and sex ratio (32%). Thus, the disadvantage of the *GFP* males in precopulatory sexual selection, at least under this setting, was large enough to explain the degree of their disadvantage in total sexual selection measured by paternity.

## DISCUSSION

We found that the operational sex ratio had major effects on the strength of sexual selection against two mutant alleles, with the relative sexual fitness of their homozygous carries ranging from about 1 to 0.5 for *ebony* and from 0.8 to 0.3 for *GFP*. However, the patterns of this effect differed between the mutants: while the relative sexual fitness of the *ebony* males declined monotonically with male:female OSR, that of the *GFP* males was lowest at 1:1 OSR, except at the highest density, where it also declined monotonically with OSR. Thus, the density of the mating pool had a major effect on the relationship between OSR and the relative fitness of the *GFP* insertion, but no such effects were seen for the *ebony* mutant (except for a minor effect on day 3). The strength of sexual selection against *ebony*, as reflected in the paternity of offspring produced on the three consecutive days of the experiment, declined over the course of the experiment, whereas for the *GFP* the day of the experiment showed a complex pattern of interaction with density and OSR. Both male and female numbers contributed similarly to the effects of OSR on the strength of selection. These effects were thus not driven by changes in the absolute number of one sex but by their numbers relative to each other.

Thus, our results support the notion that OSR is a major factor that affects the strength of sexual selection, but they do not support the common assumption that sexual selection generally becomes stronger as the OSR becomes increasingly male-biased. Furthermore, they demonstrate that, even in the same species and under the same environmental conditions, the relationship between sexual selection and OSR and other factors may depend on the focal genotype and, by extrapolation, may vary among traits subject to sexual selection. This is perhaps not surprising, given the multifaceted nature of sexual selection in *D. melanogaster*, involving male–male aggression (e.g., Baxter et al. 2018), mate choice based on a combination of visual, acoustic, tactile, olfactory, and gustatory cues (e.g., Ferveur 2010; Baxter et al. 2018), sperm competition (e.g., Lupold et al. 2020) and pheromonal tug of war over female remating and short-term egg production (Holland and Rice 1999; Wigby et al. 2020; Kohlmeier et al. 2021). A male’s aptitude for these different facets of competition for female gametes is likely to be mediated by different traits, affected in part by different genes. The relative importance of each of those facets in determining a male’s reproductive success may be differentially affected by the sex ratio and other variables of the mating environment (Billeter et al. 2012). It is likely a combination of such differential responses of different components of sexual success that is responsible for the complex pattern of sexual selection against the *GFP* mutant, and a better understanding of this result would require a detailed dissection of these traits.

While the density of the mating pool did affect the relative fitness of *GFP* males at the 2:1 sex ratio, we did not see any general or consistent effect of the density of the mating pool on the strength of sexual selection against the two mutant alleles. Such effects might be expected because density affects the frequency but also likely the nature of interactions between individuals. For example, females seem to be able to exercise mate choice more effectively under a lower density of interacting individuals, presumably enhancing the importance of male attractiveness relative to aggressiveness and sperm competition ability (Yun et al. 2018). It should be noted, however, that even the lowest of our densities is quite high compared to what *Drosophila* may encounter in nature; furthermore, in contrast to nature, females could not get away or hide from males. On the other hand, even the highest density of 54 individuals per box was well below the typical density of several hundred flies per 150–200 mL bottle under standard conditions of *Drosophila* husbandry in the lab, under which our populations evolved. It is thus also possible that decades of evolution under such extreme densities led to erosion of traits (such as male ability to locate females or female choosiness) that are of little relevance in crowded lab cultures but that could have changed the outcome of sexual selection under lower densities. Thus, we believe that our results do not allow us to discount the possibility that a broader range of densities, in particular below those used in our study, would have major effects on the strength of sexual selection against deleterious alleles. Our main motivation for the density treatment was to test whether the effects are really driven by the ratio of the two sexes versus being mediated by the density of one sex.

The dynamics of sexual selection are also likely to change over time as both sexes become more experienced, males deplete their stock of sperm and seminal fluid proteins (Sirot et al. 2009), and females become less receptive and more choosy (Kohlmeier et al. 2021). Thus, the focus of sexual selection presumably shifts from the initial scramble for highly receptive females at the beginning of the experiment, to sperm competition and its avoidance later on. In this context, it is intriguing that *ebony* males seem mainly inferior in precopulatory sexual selection (Kyriacou 1981), but their sperm competition ability—at least sperm “offense”—does not seem to be significantly impaired (Hollis et al., 2019). In contrast, the *GFP* males are impaired in both sperm competition (Manier et al., 2010) and, as we have shown here, in precopulatory sexual selection. It is thus tempting to speculate that this difference may have contributed to the fact that sexual selection against *ebony* became much weaker after the first day, whereas sexual selection against the *GFP* transgene remained strong throughout the experiment. Obviously, a convincing test of this idea would require multiple mutants with impairments of specific aspects of sexual competitiveness.

Finally, we cannot exclude that part of the difference of the pattern between mutant alleles was mediated by the female genotype. Because the *ebony* phenotype is recessive but *GFP* dominant, we used *ebony* females in the *ebony* experiment but wild-type females in the *GFP* experiment (otherwise, the genetic background was the same). The molecular effects of *ebony* mutation on biogenic amine synthesis and pigmentation are not sex-specific (Wittkopp et al. 2002), and thus *ebony* females are likely to have altered visual perception, circadian rhythm, and cuticular hydrocarbons (Massey et al. 2019). Such changes might affect female choosiness or stamina in resisting male harassment in ways that affect the strength of sexual selection. Females from the IV genetic background homozygous for the ebony mutation have in the past been shown to both weigh more and lay more eggs than wild type IV females (Houle and Rowe 2003); they also appear more susceptible to mating-induced male harm (Hollis et al. 2019). Furthermore, females of the *ebony* population have several hundred generations of evolutionary history of being courted by, choosing and mating with *ebony* males, possibly resulting in evolutionary changes in their behavioral or physiological responses to them as mates. Such differences in female traits, whether caused by the mutation itself, or by changes in other genes that the mutation favored, might also have contributed to the differences in the pattern of sexual selection against the two mutants.

How representative are these two mutations of deleterious mutations envisioned by the genic capture theory as the main source of genetic variation on which sexual selection acts (Rowe and Houle 1996)? We chose them for practical reasons—they allow an easy scoring of paternity, and they have rather large effects on performance, granting reasonable statistical power. While *ebony* is a natural spontaneous mutation, the *GFP* insert is a transgene not found in nature. And, given their large effects on sexual fitness, they could hardly be described as mildly deleterious. However, some studies (reviewed in Halligan and Keightley 2009) report the mean effects of spontaneous deleterious mutations in *Drosophila* on viability or fitness to be of similar magnitude to those mediated by our mutations. Furthermore, the contribution of deleterious mutations to genetic load under mutation-selection balance is independent of their effect. Thus, mutations with such large effects cannot be considered irrelevant to sexual selection. Furthermore, at least the *ebony* mutation has the highly pleiotropic nature envisioned by Rowe and Houle (1996) for mutations affecting an animal’s general somatic condition. It perturbs a major metabolic pathway with phenotypic effects ranging from coloration, through visual perception, to behavior and life history (Kyriacou 1985; Wittkopp et al. 2002; Houle and Rowe 2003; Richardt et al. 2003; Kawecki 2020). The degree of pleiotropy of the *GFP* insertion is less clear. It had been known to impair sperm competitive ability, presumably because expression of the green fluorescent protein in the sperm imposes a cost on the highly strung resources of the sperm cell. We initially assumed that it would not impair competition for mates, but as our results show, it clearly does, possibly simply as a consequence of the organism wasting resources to produce a useless protein. Such costs would likely affect non-sexual aspects of fitness. Thus, both alleles would fit the bill of “bad genes/good genes” polymorphisms thought to fuel the genetic benefits of female choice (Rowe and Houle 1996).

In spite of the popularity of the notion that sexual selection on male traits should become monotonically stronger with increasing male:female OSR, our experiment with *ebony* appears to be the only one that demonstrated this relationship experimentally by quantifying sexual selection under OSR ranging from female-biased through 1:1 to male-biased. A similar monotonic trend has been reported for sexual selection on male body size in pipefish, but without a statistical test that would support it (Jones et al. 2005). Sexual selection on two sexual ornaments was also reported to be stronger in 1:1 than in female-biased sex ratio in a goby, but male-biased sex ratios were not included in the design (Wacker et al. 2013). However, two studies with bank voles suggest that sexual selection in that system is strongest at intermediate OSR and vanishes in highly male-biased OSR. One study (Mills et al. 2007) reported sexual selection gradients on testosterone levels that were intermediate at 1:1 sex ratio, highest at 3:2 male:female OSR but became essentially zero in 2:1 OSR. Similarly, in an independent experiment, sexual selection on male body size was similarly strong at 2:5 and 1:1 OSR (with the point estimate higher in the latter) but vanished at 5:1 OSR (Klemme et al. 2007). Neither study tested statistically for the nonlinear nature of this apparent relationship. Nonetheless, those studies increase our confidence that the weakening of sexual selection on the *GFP* transgene polymorphism at 2:1 male-biased sex ratio may not be a rare exception or an artifact of using an “unnatural” mutation.

If so, male-biased OSRs would reduce the effectiveness of sexual selection in purging some deleterious mutations (Cally et al. 2019). Furthermore, from the viewpoint of females, male-biased OSR would not only result in an increased male harm due to harassment and pheromonal manipulation, but it would also reduce the females’ chances to obtain “good genes” for their offspring. This form of (interlocus) sexual conflict that restricts the female’s ability to mate with the optimal mate would thus contribute to the selection of females to avoid mating in highly male-biased aggregations (Snow et al. 2019).

## Supplementary Material

arad110_suppl_Supplementary_Figures_S1-41467_Tables_S1-S6

## Data Availability

Analyses reported in this article can be reproduced using the data provided by Sharda et al. (2023).
