# Supplementary material for: Sex ratio affects sexual selection against mutant alleles in a locus-specific way
Source: Behav Ecol. 2023 Dec 29;35(1):arad110. doi: 10.1093/beheco/arad110 (PMC10756055; doi:10.1093/beheco/arad110)
Supplement: arad110_suppl_Supplementary_Figures_S1-41467_Tables_S1-S6 [file arad110_suppl_supplementary_figures_s1-41467_tables_s1-s6.pdf]

Sex ratio affects sexual selection against mutant alleles in a locus-specific way

Sakshi Sharda[1], Brian Hollis[2], Tadeusz J. Kawecki[1,3]

[1] Department of Ecology and Evolution, University of Lausanne, CH-1015 Lausanne, Switzerland

[2] Department of Biological Sciences, University of South Carolina, Columbia SC 29208, USA

[3] Corresponding author; tadeusz.kawecki@unil.ch

Supplementary Figures and Tables

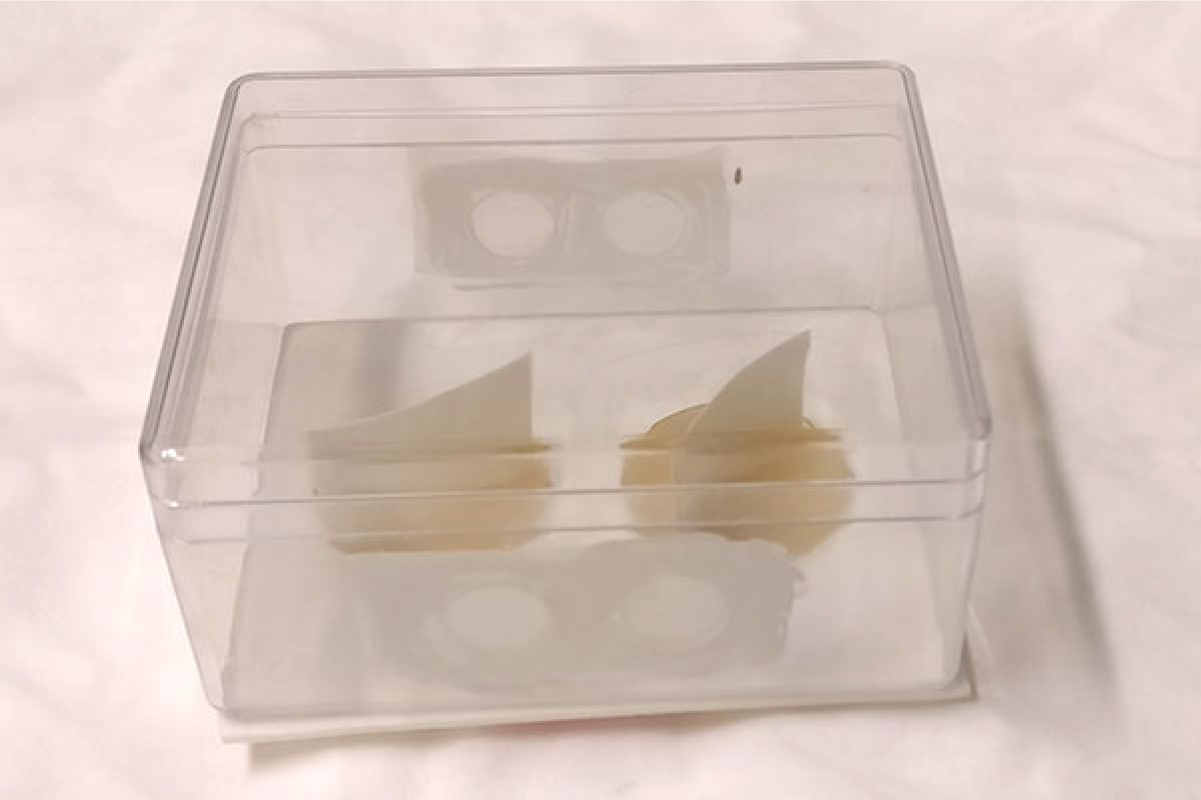

Supplementary Figure S1. The cage used for the experiments. A polystyrene box (100 × 85 × 46 mm) with two replaceable circular inserts for petri-dishes standard fly food or medium for collecting eggs (agar + orange juice sprinkled with Baker's yeast). The partition on the petri-dishes adds a component of spatial complexity.

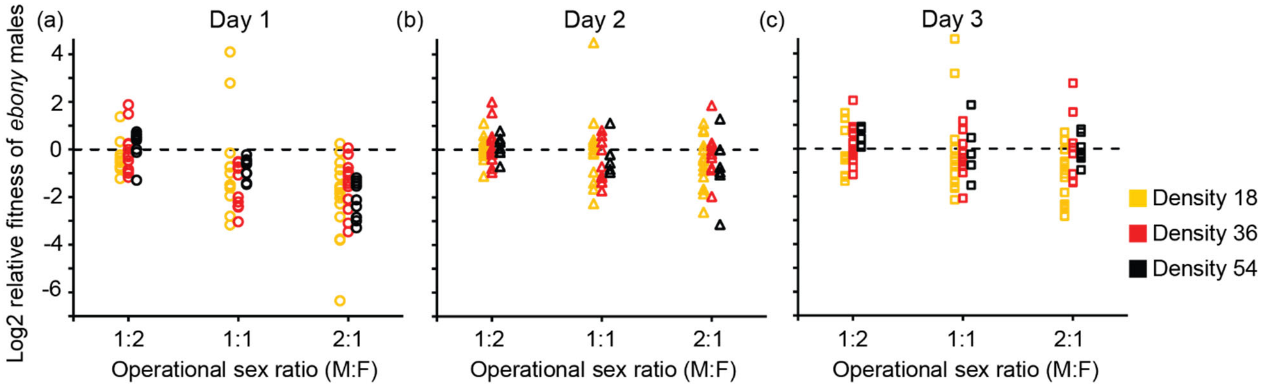

Supplementary Figure S2. Data underlying Figure 1 plotted as estimates of relative sexual fitness of *ebony* males from each replicate cage, depending on the operational sex ratio and density, on the three consecutive days of the experiment.

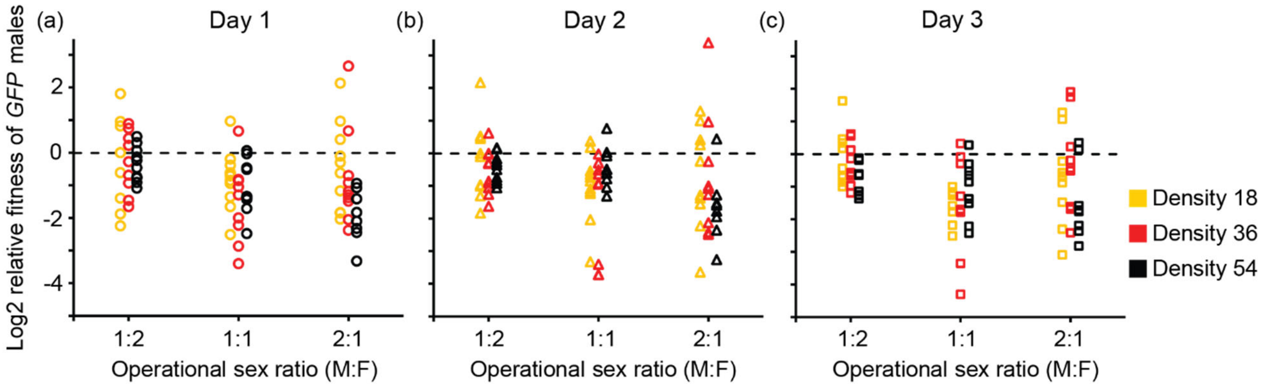

Supplementary Figure S3. Data underlying Figure 2 plotted as estimates of relative sexual fitness of *GFP* males from each replicate cage. Note that plots are grouped by day rather than by density as in Figure 2.

Supplementary Table S1. The number of replicate paternity estimates for each treatment (density × OSR combination). Although the same replicate cages were used to obtain paternity estimates on the three consecutive days of the experiment, the number vary across days because some estimates were not obtained for diverse reasons (see Methods).

Density	OSR	Number of replicates		
		Day 1	Day 2	Day 3
ebony				
18	1:2	10	11	11
18	1:1	13	13	13
18	2:1	16	15	15
36	1:2	12	14	15
36	1:1	11	11	11
36	2:1	13	10	8
54	1:2	8	9	8
54	1:1	8	6	5
54	2:1	9	7	8
GFP				
18	1:2	8	9	9
18	1:1	11	11	10
18	2:1	10	10	10
36	1:2	9	10	10
36	1:1	9	9	9
36	2:1	10	10	10
54	1:2	10	10	10
54	1:1	9	9	9
54	2:1	8	8	8

Supplementary Table S2. GLMM (logit link, binomial distribution) for the paternity (number of offspring sired) by *ebony* males relative to wildtype males. Parameters and their standard errors are on the logit scale; the χ^2 (always df = 1) and *P* refer to the likelihood ratio test.

Factor	Parameter	SE	χ^2_1	P
OSR	-0.540	0.069	52.1	**< 0.0001**
Density	-0.021	0.071	0.1	0.76
Day	0.301	0.025	148.3	**< 0.0001**
OSR × Density	-0.147	0.086	2.9	0.09
OSR × Day	0.218	0.029	55.4	**< 0.0001**
Density × Day	0.117	0.031	14.1	**0.0002**
OSR × Density × Day	0.117	0.036	10.5	**0.0012**
Analysis split by day[1]				
Day 1:				
OSR	-0.569	0.088	35.1	**< 0.0001**
Density	0.029	0.092	0.1	0.75
Day 2:				
OSR	-0.230	0.077	8.5	**0.0036**
Density	0.001	0.084	0.0	0.99
Day 3:				
OSR	-0.195	0.085	5.1	**0.024**
Density	0.256	0.091	7.5	**0.0063**

[1]OSR × Density interaction was not significant for any day (all *P* > 0.25) and was removed from the model

Supplementary Table S3. GLMM for the paternity success of *ebony* males as in Table S1 but, additionally including the total number of offspring as a covariate.

Factor	Parameter	SE	χ^2_1	P
OSR	-0.535	0.069	50.8	**< 0.0001**
Density	-0.021	0.071	0.1	0.77
Day	0.300	0.025	146.4	**< 0.0001**
OSR × Density	-0.149	0.087	3.0	0.086
OSR × Day	0.215	0.030	53.6	**< 0.0001**
Density × Day	0.118	0.031	14.4	**0.0002**
OSR × Density × Day	0.117	0.036	10.4	**0.0013**
Total offspring	-0.030	0.026	1.3	0.25
Analysis split by Day				
Day 1:				
OSR	-0.571	0.090	34.7	**< 0.0001**
Density	0.029	0.092	0.1	0.75
Total offspring	0.011	0.065	0.0	0.87
Day 2:				
OSR	-0.194	0.077	6.2	**0.01**
Density	0.014	0.080	0.0	0.86
Total offspring	-0.190	0.076	5.9	**0.01**
Day 3:				
OSR	-0.205	0.084	5.8	**0.016**
Density	0.241	0.090	6.8	**0.0090**
Total offspring	0.142	0.076	3.4	0.064

Supplementary Table S4. GLMM for the paternity success of *ebony* males in the alternative reference frame, i.e., with male and female density instead of OSR and total density as explanatory variables.

Factor	Parameter	SE	χ^2_1	P
Male density	-0.526	0.085	34.1	**< 0.0001**
Female density	0.499	0.085	31.1	**< 0.0001**
Day	0.317	0.028	131.6	**< 0.0001**
Male density × Female density	-0.074	0.190	0.2	0.70
Male density × Day	0.279	0.039	51.7	**< 0.0001**
Female density × Day	-0.166	0.039	18.3	**< 0.0001**
Male density × Female density × Day	-0.118	0.091	1.7	0.20

Supplementary Table S5. GLMM (logit link, binomial distribution) for the paternity (number of offspring sired) by GFP males relative to wildtype males. Parameters and their standard errors are on the logit scale; the χ^2 (always df = 1) and *P* refer to the likelihood ratio test.

Factor	Parameter	SE	χ^2_1	P
OSR	-0.077	0.075	1.0	0.31
Density	-0.034	0.081	0.2	0.67
Day	-0.002	0.021	0.0	0.94
OSR × Density	-0.257	0.096	7.0	**0.0080**
OSR × Day	0.022	0.026	0.7	0.40
Density × Day	0.009	0.026	0.1	0.72
OSR × Density × Day	0.136	0.032	17.8	**< 0.0001**
Analysis split by density				
Density = 18				
OSR	0.012	0.144	0.0	0.93
Day	-0.231	0.060	14.9	**0.0001**
OSR^2	0.130	0.235	0.3	0.58
OSR × Day	-0.138	0.046	9.0	**0.0026**
OSR^2 × Day	0.275	0.076	13.2	**0.0003**
Density = 36				
OSR	0.019	0.149	0.0	0.90
Day	0.097	0.037	6.9	**0.0088**
OSR^2	0.490	0.241	3.9	**0.047**
OSR × Day	0.066	0.045	2.2	0.14
Density = 54				
OSR	-0.541	0.094	22.5	**< 0.0001**
Day	-0.039	0.036	1.2	0.28
OSR^2	-0.218	0.139	2.3	0.13
OSR × Day	0.119	0.046	6.8	**0.0092**

Supplementary Table S6. GLMM for the paternity success of the GFP males in the alternative reference frame, i.e., with male and female density instead of OSR and total density as explanatory variables.

Factor	Parameter	SE	χ^2_1	*P*
Male density	-0.153	0.078	3.8	0.052
Female density	0.140	0.095	2.1	0.15
Day	0.030	0.025	1.5	0.22
Male density × Female density	0.153	0.215	0.5	0.48
Male density × Day	0.043	0.031	1.9	0.17
Female density × Day	-0.076	0.029	6.9	**0.0090**
Male density × Female density × Day	-0.175	0.068	6.6	**0.010**
